# NMR Spectroscopic Characterization of the C‐Mannose Conformation in a Thrombospondin Repeat Using a Selective Labeling Approach

**DOI:** 10.1002/anie.202009489

**Published:** 2020-09-03

**Authors:** Hendrik R. A. Jonker, Krishna Saxena, Aleksandra Shcherbakova, Birgit Tiemann, Hans Bakker, Harald Schwalbe

**Affiliations:** ^1^ Institute for Organic Chemistry and Chemical Biology Center for Biomolecular Magnetic Resonance (BMRZ) Goethe University Frankfurt Max-von-Laue Strasse 7 60438 Frankfurt am Main Germany; ^2^ Institute of Clinical Biochemistry Hannover Medical School Carl-Neuberg-Strasse 1 30625 Hannover Germany

**Keywords:** C-mannosylation, conformation analysis, NMR spectroscopy, selective isotopic labeling, glycoproteins

## Abstract

Despite the great interest in glycoproteins, structural information reporting on conformation and dynamics of the sugar moieties are limited. We present a new biochemical method to express proteins with glycans that are selectively labeled with NMR‐active nuclei. We report on the incorporation of ^13^C‐labeled mannose in the C‐mannosylated UNC‐5 thrombospondin repeat. The conformational landscape of the C‐mannose sugar puckers attached to tryptophan residues of UNC‐5 is characterized by interconversion between the canonical ^1^C_4_ state and the B_03_ / ^1^S_3_ state. This flexibility may be essential for protein folding and stabilization. We foresee that this versatile tool to produce proteins with selectively labeled C‐mannose can be applied and adjusted to other systems and modifications and potentially paves a way to advance glycoprotein research by unravelling the dynamical and conformational properties of glycan structures and their interactions.

## Graphical abstract

NMR spectroscopy was used to characterize the C‐mannose conformation in a thrombospondin repeat using a selective labeling approach. The conformational landscape of the C‐mannose sugar puckers attached to tryptophan residues of the netrin receptor UNC‐5 allows interconversion between the canonical ^1^C_4_ state and the B_03_ / ^1^S_3_ state.

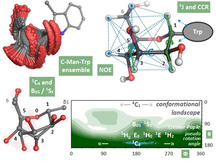

## Introduction

Glycosylation plays an essential role in the structure and biological function of many proteins in living cells and viruses.^[1]^ The process that modifies proteins with glycans is highly complex and tightly regulated by the specific cellular microenvironment and orchestrated by numerous glycosyltransferases and glycosidases.^[2]^ Glycan structures and the glycosylation patterns are highly dynamic and change during development. Even though more than half of all human proteins are glycosylated, the functional characteristics of this important post‐translational modification is so far poorly understood and structural information is scarce. On the molecular basis it is essential to understand how the linked sugars can impact the stability, structure and function of glycoproteins. These small key elements are very diverse and can exist in many conformations. The different shapes of the six‐membered sugar ring can be described by 38 canonical states.[^3^, ^4^, ^5^] Theoretical studies indicated the importance of the sugar ring flexibility and gave insight in the interconversion pathways between the different geometries.[^6^, ^7^] For particular glycans a distortion of the pyranose ring may be necessary for biomolecular interactions, which makes characterization of the sugar pucker and its dynamics essential to understand the feasible selection of the specific conformations. Unfortunately, due to the flexibility of the glycans and incomplete glycosylation, the relevant electron density is often limited or even lacking in X‐ray crystallography studies, and therefore the sugar moieties of the glycoproteins are frequently missing or modelled. Also, structural investigations by Nuclear Magnetic Resonance (NMR) has its limitations due to the severe overlap of the proton signals between the carbohydrate and the protein. This can be partially resolved by the rather difficult and often less efficient eukaryotic isotope labelling[^8^, ^9^, ^10^, ^11^] or in vitro glycosylation technologies.^[12]^ These challenges need to be tackled and the current methods need to be further refined to realize more detailed and straightforward structure elucidation of glycoproteins.

Among the many types of glycosylation, C‐mannosylation (C‐Man) of a tryptophan residue is a unique post‐translational modification which occurs in a variety of proteins. A single mannose is distinctively attached to the tryptophan indole sidechain via a carbon‐carbon linkage between the anomeric sugar C_1_ carbon and the C_δ1_ carbon atom of the amino acid side chain. C‐Man was first reported in human ribonuclease 2^[13]^ and occurs at specific sites in many proteins (such as the cytokine type I receptors and thrombospondin type 1 repeats (TRSs). C‐Man is catalyzed by enzymes of the C‐ManT family in the endoplasmic reticulum (ER) membrane using dolichol‐P‐mannose as a donor substrate^[14]^ and usually occurs at the first tryptophan in the consensus sequence motif WXXW/C of the protein. There are two C‐ManTs (DPY19L1 and ‐3) in mammals with distinct specificities towards their protein substrate consensus motifs and two other putative C‐ManTs (DPY19L2 and ‐4).^[15]^

Considering the important role of glycans in protein function, the development of methods to produce and analyze glycosylated proteins is essential. Glycosylation is often incomplete and the proteins and glycans are frequently very dynamic and exhibit a high degree of conformational heterogeneity. Yet, solution NMR spectroscopy is particularly well suited for the analysis and structural studies of such inhomogeneous and complicated targets.^[16]^ Moreover, the rich diversity and the complex equilibria of interconverting forms of saccharides and the structural complexity of the glycan composition can be characterized in detail by NMR.[^17^, ^18^, ^19^, ^20^] Here we describe a new eukaryotic expression strategy to prepare selective ^13^C‐isotope labeled C‐Man glycoproteins; which allows specific investigation of the glycans present in proteins by solution NMR spectroscopy. This previously unknown labeling technique is based on the development of a *Drosophila* S2 cell line that cannot use glucose to generate mannose and are therefore dependent on the supplied mannose. The approach has been applied here to characterize the conformational dynamics of the C‐α‐mannose moieties of a thrombospondin repeat from the netrin receptor UNC‐5, which is involved in axon guidance by repulsion.[^21^, ^22^, ^23^] Our study showcases the information that can be obtained with this methodology to advance the structural biology of glycosylated proteins.

## Results and Discussion

### Production of Selective ^13^C‐mannose Labeled UNC‐5 Protein

**Figure 1:**
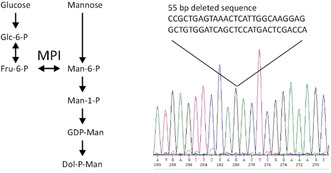
Development of a *Drosophila* S2 cell line that cannot use glucose to generate mannose. Knock‐out of the enzyme mannose phosphate isomerase (MPI) by homozygous deletion of 55 bp in the gene sequence (right) disables the conversion of fructose‐6‐phosphate into mannose‐6‐phosphate (left).

A *Drosophila* S2 cell line, stably expressing a single thrombospondin type 1 repeat (TSR) of the netrin receptor UNC‐5, has been generated and described before.^[24]^ Co‐expression of the C‐mannosyltransferase DPY‐19 from *C. elegans* allowed producing the C‐mannosylated form of the repeat. The TSR2 of UNC‐5 starts with the sequence LDGGWSSWSDWSAC, in which the first two tryptophans can be mannosylated by DPY‐19^[25]^ and are named W5 and W8 here (W277 and W280 in the original sequence, uniprot: Q26261). To be able to selectively label mannoses in this cell line, we introduced a mutation in the enzyme mannose phosphate isomerase (MPI) by CRISPR‐CAS. MPI is responsible for the reversible conversion of fructose‐6‐phosphate into mannose‐6‐phosphate (Figure 1), which enables cells to use glucose as a source for the mannoses found in glycoproteins. On the other hand, exogenous or salvaged mannose can also be converted in other monosaccharides and used for glycolysis. Knock‐out the MPI enzyme thus resolves two problems in one. The isotope label of ^13^C‐mannose cannot end up in other metabolites and the ^13^C‐mannose pool is not diluted.

The generated MPI knock‐out clone has a homozygous deletion of 55 bp in the gene sequence (Figure 1). We established that the generated cell line was not able to grow permanently without added mannose, and that normal growth was supported over a range of 10 μm to 1 mm exogeneous mannose. The clone was routinely maintained in medium with 100 μm added mannose. To produce selective ^13^C‐labeled mannose in the UNC‐5 protein cells were cultured at a 300 mL scale and supplied with 500 μm ^13^C‐isotope labeled mannose. Two mg of highly pure UNC‐5 TSR2 was produced that uniformly contained two C‐mannoses (details in the supporting information experimental section).

### NMR Analysis

**Figure 2:**
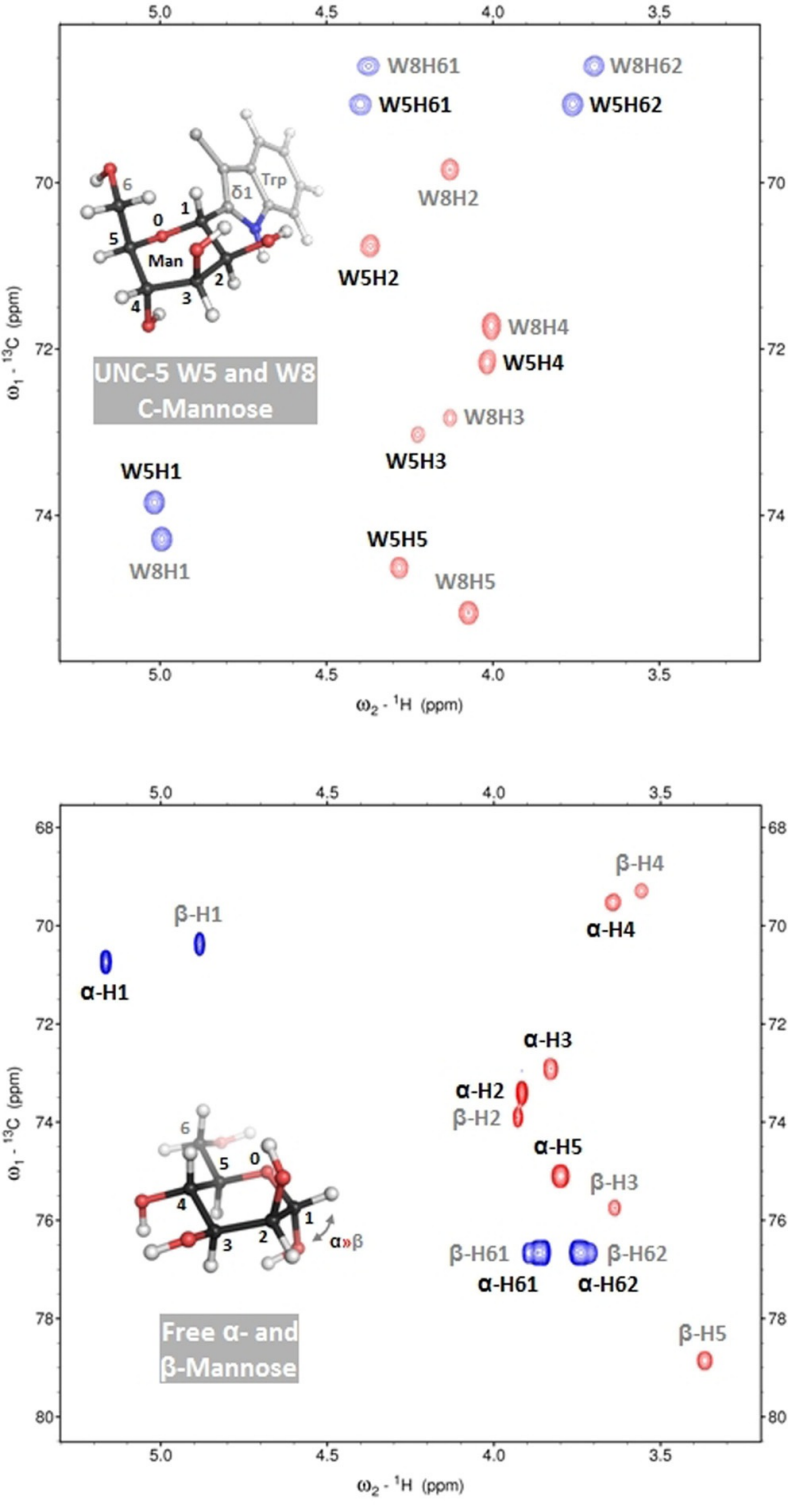
Incorporation of selective ^13^C‐labeled mannose in the UNC‐5 protein. The ^1^H^13^C‐CT‐HSQC spectrum (on the top) shows the mannose sugar CH correlation signals of the selective ^13^C‐labeled C‐mannoses that are covalently attached to tryptophans 5 and 8 in the UNC‐5 protein (1.25 mm) measured at 600 MHz and a temperature of 298 K. For optimal acquisition time and resolution, the signals of C_1_H_1_, C_5_H_5_, and C_6_H_6#_ are folded in the carbon dimension (spectral width of 7.7 ppm) and actually appear around 66.4, 82.6, and 61.1 ppm, respectively. The inserted structure shows a C‐α‐mannose moiety (in a ^1^C_4_ conformation) connected to a tryptophan (mannose C_1_ to tryptophan C_δ1_) and indicates the numbering of the observed signals in the NMR spectrum. The ^1^H^13^C‐CT‐HSQC spectrum of free ^13^C‐labeled mannose (on the bottom) shows the sugar CH correlation signals of the α and β conformation (anomeric mixture, 10 mm) likewise measured at 600 MHz and a temperature of 298 K. For optimal acquisition time and resolution, the signals of C_1_H_1_ and C_6_H_6#_ are folded in the carbon dimension (spectral width of 13.0 ppm) and actually appear around 96.6 and 63.7 ppm, respectively. The inserted structure shows a free α‐mannose (in a ^4^C_1_ conformation) and indicates the numbering of the observed signals in the NMR spectrum.

The successful incorporation of selective ^13^C‐labeled mannose in the UNC‐5 protein is shown in the ^1^H^13^C‐HSQC spectrum (Figure 2). Both mannose moieties of the C‐Man tryptophans W5 and W8 show distinct chemical shift resonances. Advanced NMR experiments (among others ^13^C‐resolved 3D and 4D NOESY, quantitative Γ‐HCCH and heteronuclear ^13^C‐relaxation) were performed on this selective ^13^C‐isotope labeled probe to determine the exact sugar geometry and dynamics. Theoretical puckering studies exploring the kinetic energy landscape for several sugars shows that there are different favorable geometries and many possible interconversion pathways.[^6^, ^7^] The various shapes and distortions of the sugar moiety are influenced by *exo*‐anomeric effects and the type of glycosidic linkage.[^26^, ^27^] Moreover, catalysis mechanisms typically take advantage of the pyranose ring flexibility and proceed through distinct transition state conformations.^[28]^ Hence, investigation of the puckering landscape is crucial to understand which particular geometries are favored, especially within the complexity of glycobiology.

### Dynamics

In order to probe the dynamical behavior of the mannose moieties, heteronuclear ^13^C‐relaxation experiments (for the longitudinal ^13^C‐T_1_ and off‐resonance rotating frame ^13^C‐T_1ρ_ relaxation rates and {^1^H}^13^C‐heteronuclear NOE) were performed on the selective labeled UNC‐5 protein sample as well as on free mannose, under the same conditions. Due to uniform isotope labelling of the sugar moiety, direct determination of ^13^C‐T_2_ is not possible. Therefore, the transverse ^13^C‐T_2_ relaxation rates were extracted from off‐resonance correction of the T_1ρ_ values using T_1_. As expected, the rates and HetNOE values (supplementary Table 1) are clearly very different for the free and the covalently attached mannose, which correlates to the differences in their overall tumbling rate as well as their mobility. To evaluate the dynamical behavior, the relaxation data were analyzed using the model‐free method.[^29^, ^30^] The free mannose (both α and β) is highly dynamic, and the overall rotational correlation time *τ*_c_ is very fast (about 125 ps), making separation of the overall motion and internal motion complicated and thus practically unfeasible. However, when the mannose moiety is covalently attached to the tryptophan, the dynamics are strongly reduced. The average order parameter for the C‐Man W5 is 0.91 and for the C‐Man W8 0.94, indicating that those moieties become rigidified when attached and reside within the environment of the UNC‐5 protein. Furthermore, the model‐free analysis points to the presence of little internal dynamics (*τ*_e_ in the order of about 60 ps) for the slightly more dynamic C‐Man W5.

### Conformation by ^3^ *J*(H,H) Couplings

**Figure 3:**
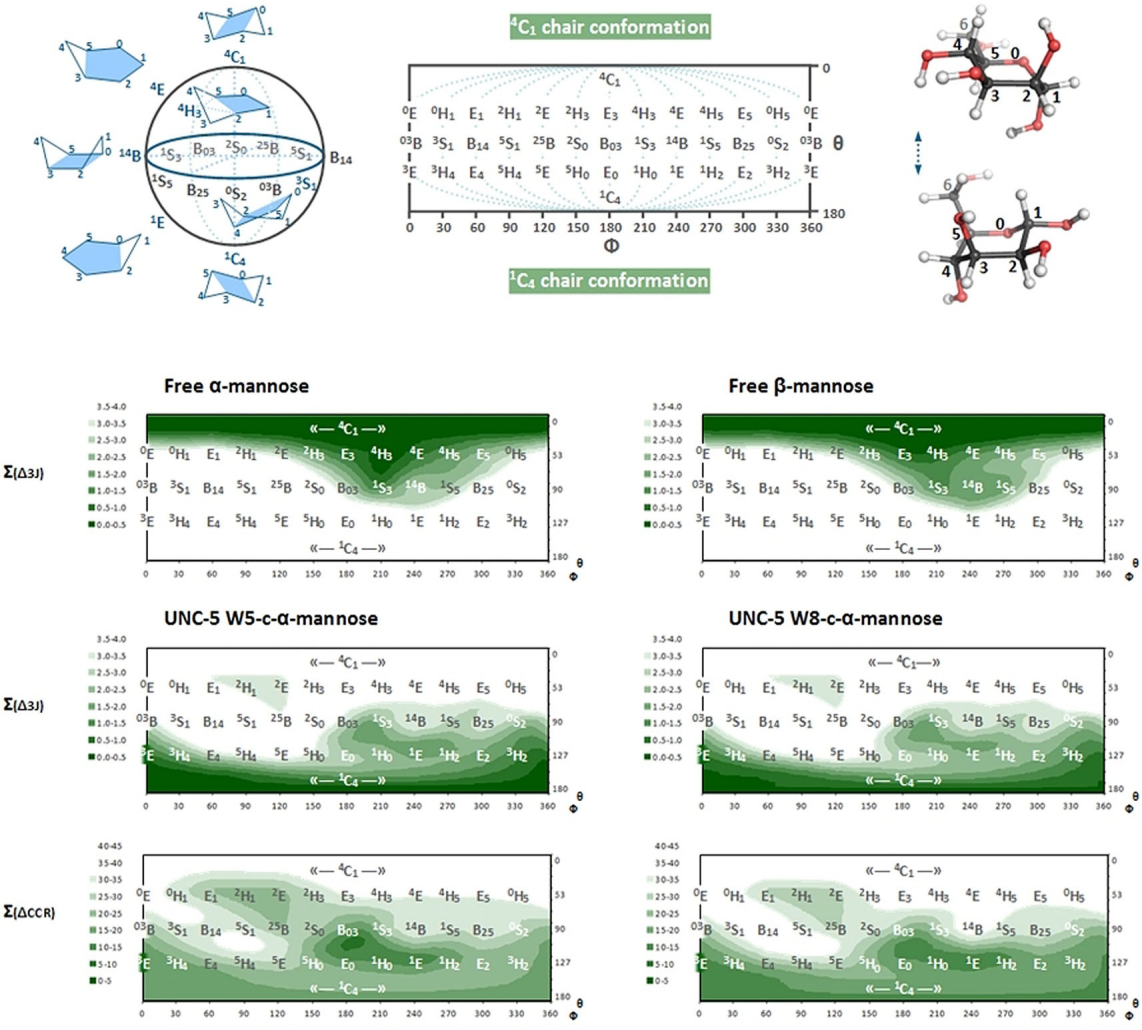
Exploring the landscape of mannose sugar conformations. The 38 canonical ring puckering designations (2 chair (C), 12 envelope (E), 12 half‐chair (H), 6 boat (B), 6 skew (S) ) are illustrated (on top) by a 2D mercator projection map of the “Cremer–Pople” sphere.^[5]^ The pictures are based on and adapted from Hill and Reilly,^[4]^ Bérces et al.^[3]^ and Mayes et al.^[7]^ The combined differences for either ^3^ *J*(H,H) and CCR(CH,CH) values are indicated below for free mannose and UNC‐5 C‐mannose, illustrating the regions of favorable sugar geometries. For each conformation the difference of the experimental values from the theoretical expected values (Karplus equation) was calculated and combined according to: ∑Δ=0.5√(Δ^2^_H1H2_+Δ^2^_H2H3_+Δ^2^_H3H4_+Δ^2^_H4H5_). The ^3^ *J*(H,H) couplings of free mannose (both α and β anomers) indicate a conformation in the ^4^C_1_ hemisphere; while the conformation of the UNC‐5 C‐mannose (both W5 and W8) spans a likely range in the ^1^C_4_ hemisphere, which is also observed by the same analysis for CCR(CH,CH). The separate analysis for free mannose and the UNC5‐C‐Man W8 and a table of the experimental values for each ^3^ *J*(H,H) coupling or CCR(CH,CH) is given in the supplementary information.

The conformation of the mannose six‐membered ring moiety was first probed by determination of ^3^ *J*(H,H), the vicinal proton‐proton coupling constants. The dependence of the individual expected couplings on the corresponding endocyclic torsion angles of the pyranose conformations^[3]^ can be approximated well using the generalized Karplus equation including a correction for the electronegativity of the substituents.^[31]^ For these individual couplings, several conformations are often possible, which cannot be distinguished due to the complex landscape for the pyranose ring and the presence of multiple minima in the Karplus relation (supplementary Figures 1 and 2). Nevertheless, a combination of the coupling constants can characterize the ring puckering. Therefore, we jointly analyzed the results from all couplings and map their combined difference on the 2D mercator projection of the “Cremer–Pople” sphere (Figure 3). The combined difference plot substantially restricts the regions of the conformational landscape in accordance with the experimental data.

For both free α‐mannose as well as β‐mannose, the analysis of the ^3^ *J*(H,H) couplings indicates that those are mostly present in the ^4^C_1_ conformation (and possibly fluctuate to adjacent conformations such as E_3_ and ^4^H_3_). In contrast, the C‐mannose moieties of the UNC‐5 W5 and W8 mostly map towards the conformational space in the ^1^C_4_ hemisphere (including sugar pucker geometries such as B_03_ and ^1^S_3_). Remarkably, for the C‐Man W5 and W8, the observed intermediate ^3^ *J*(H_3_,H_4_) values (supplementary Figure 2 C) do not fully agree with either ^1^C_4_ or ^4^C_1_ conformation, as also observed by others.[^32^, ^33^] This result fits to prior theoretical considerations and can be explained by the preference of the bulky tryptophan, which is covalently attached to the C_1_ carbon of the mannose to be in the equatorial position. The experimental coupling may possibly be an intermediate; probing an ensemble or exchange between two (or) more conformations that deviate around the H_3_‐C_3_‐C_4_‐H_4_ torsion angle (such as the B_03_ boat and ^1^S_3_ skew).

### Conformation by CCR

For free mannose, a small molecule, the vicinal ^3^ *J*(H,H) couplings can be determined accurately, but for a covalently attached C‐mannose, which is in the context of the larger protein, the quantitative analysis becomes less accurate and less precise due to severe overlap, line broadening effects but also potential systematic errors.^[34]^ Yet, the size of measurable effect of another parameter, cross‐correlated relaxation (CCR), linearly depends on the overall rotational correlation time (*τ*_c_) and can be conveniently used for the study of larger biomolecules.[^35^, ^36^] The cross‐correlation rates report on the project angles of CH‐CH bond vectors and are geometrically uniquely related to the torsion angles that can be measured via vicinal ^3^ *J* proton‐proton couplings. The selective ^13^C‐mannose labeled UNC‐5 protein sample allowed us to determine the CCRs for the C‐mannose moieties of the UNC‐5 W5 and W8 by means of quantitative 2D ^1^H^13^C‐Γ‐HCCH experiments (supplementary Figure 3). In addition to the 2D version, a 3D experiment was exploited, adding an additional carbon dimension, to resolve signal‐overlap (such as for the W8 C‐mannose H2 and H3 protons which appear at the same resonance). The obtained CCR(CH,CH) data was analyzed in the same way as for the ^3^ *J*(H,H) coupling constants (Figure 3 and supplementary Figure 2). The combined difference landscape indicates essentially the same conformational space in the ^1^C_4_ hemisphere as observed for the vicinal coupling constants. Moreover, the experimental CCR values between C_3_H_3_ and C_4_H_4_ resemble the observation of the intermediate ^3^ *J*(H_3_,H_4_) coupling (supplementary Figure 2 C) likewise indicating possible mobility and/or existence of more conformational states that fast interconvert on the NMR timescale. The combined difference was also re‐examined without these values (to avoid the impact of this divergent torsion angle on the outcome of the analysis, supplementary Figure 4) and actually still shows analogous landscapes; clearly mapping the C‐mannose sugar geometry towards the conformational space in the ^1^C_4_ hemisphere.

### Conformation by NOE

Particularly important for biomolecular structure determination is the Nuclear Overhauser Effect (NOE), providing spatial distances between protons. For glycosylated proteins, the NOE buildup for the sugar is efficient because of the increased overall tumbling time due to the covalent attachment to the protein. The well‐established NOE can therefore be readily used to calculate and validate the geometry of the sugar. Moreover, fluctuations in the distance (due to dynamics or the presence of multiple conformational states) would be reflected in an averaged NOE. However, NOE averaging is weighted by 1/*r* ^−6^ and therefore substantially less sensitive to averaging compared to both, ^3^ *J*(H,H) couplings and CCR(CH,CH).

**Figure 4:**
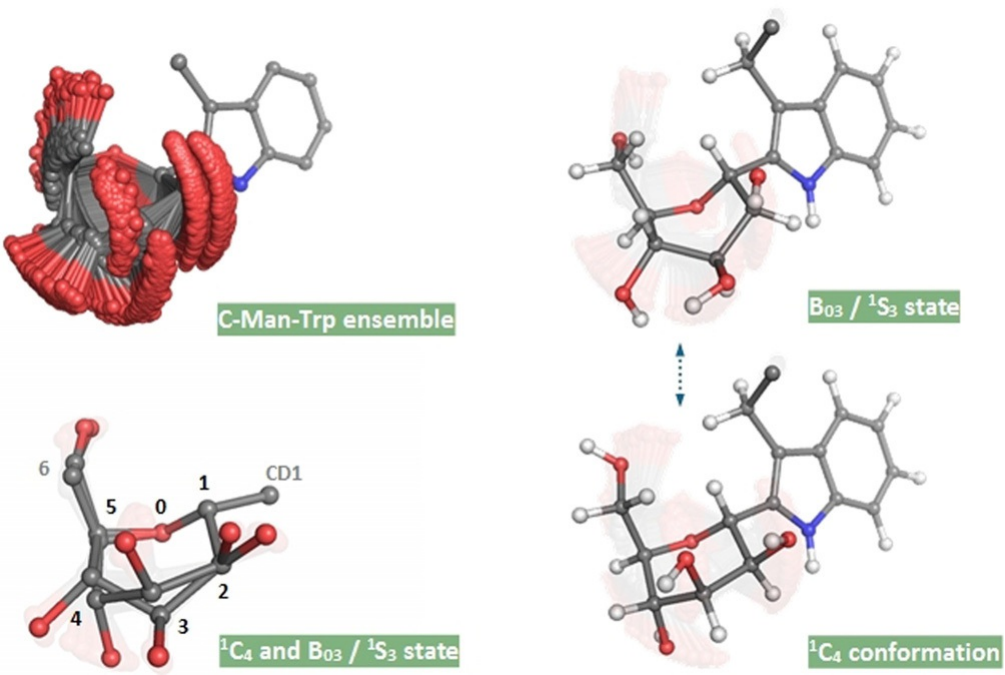
Structural space of the C‐mannosylated tryptophan. The ensemble of C‐mannosylated tryptophan structures (top left) shows a conformational space encompassing the canonical ^1^C_4_ chair and B_03_ boat / ^1^S_3_ skew conformations (right). For clarity, the hydrogen atoms are omitted in the structure pictures on the left. The divergence of the six‐membered ring (around the atoms C_3_ and C_4_) between the ^1^C_4_ and B_03_ / ^1^S_3_ geometries is indicated (bottom left) and in agreement with their experimentally observed intermediate ^3^ *J*(H_3_,H_4_) and CCR(C_3_H_3_,C_4_H_4_) values (supplementary Figure 2 C) likewise demonstrating interconverting conformational states. The structures are shown for the UNC‐5 W8 C‐mannose. The ensemble of conformations of the UNC‐5 W5 C‐mannose tends more towards the B_03_ / ^1^S_3_ geometry.

We verified whether the observed NOEs are in agreement with the C‐mannose sugar conformational space and performed a conventional structure calculation of the UNC‐5 W5 and W8 C‐mannose moieties. The UNC‐5 protein sample with ^13^C‐selective labeled mannose facilitates the use of ^13^C‐resolved NOESY experiments, which is crucial to separate overlapping NOE cross peaks. NOEs were obtained from several 3D ^1^H^1^H^13^C‐NOESY‐HSQC spectra acquired with different mixing times (NOE build‐up) and from a 4D ^1^H^13^C^1^H^13^C‐NOESY‐HSQC spectrum (to further resolve signal‐overlap) measured on the selective ^13^C‐labeled C‐Man UNC‐5 protein. From modelled structures of all 38 canonical states the expected proton‐proton distances were compared. Even though most combinations feature rather similar distances for all geometries close to the NOE distance error ranges (with only about 0.6 Ångstrom or less difference between the ^4^C_1_ and ^1^C_4_ conformation), taken together (and in combination or verification with the dihedral angles derived from ^3^ *J*(H,H) couplings and CCR(CH,CH) data) they can enclose the global shape of the sugar. The most indicative NOE to differentiate between the ^4^C_1_ and ^1^C_4_ conformation is the H_3_‐H_5_ distance (about 1.8 Ångstrom difference; expecting a strong NOE for ^4^C_1_ and weak NOE for ^1^C_4_). The NOE cross peak between these two protons is inconveniently very close to the diagonal (and for W8 strongly overlapping with H_3_‐H_4_), but this could be well resolved by using the 4D experiment. For W5, only a very weak NOE was observed between H_3_ and H_5_ and for W8 there was no cross peak detected, which is indicative for the ^1^C_4_ hemisphere and thus nicely in agreement with the previous findings. In addition, NOEs observed between the tryptophan and mannose (eg. H_β#_‐H_1_ and H_ϵ1_‐H_2_) resolve their relative orientation. The structure of the C‐Man tryptophan moieties was calculated using ARIA/CNS.[^37^, ^38^, ^39^] The bundle of calculated structures (Figure 4) encompasses a rather diffuse spatial area for the mannose. This result is expected, as the structure relies solely on intra‐residue restraints and is not further restricted and affected by sequential, medium and long range NOEs. Nevertheless, the network of NOE distance restraints limits the sugar pucker conformational space mostly to a mixture of the ^1^C_4_ chair and a state adjoining the B_03_ boat and ^1^S_3_ skew geometry (where the W5 C‐mannose structure calculations converge little more towards the B_03_ / ^1^S_3_ state, supplementary Figure 4). This NOE based finding is well in agreement with the conformational landscapes indicated by the ^3^ *J*(H,H) couplings and CCR(CH,CH) data (Figure 3) and the intermediate experimental values observed for H_3_‐H_4_. Our data thus indicates that the C‐mannose puckers of the UNC‐5 thrombospondin repeat are mostly in the ^1^C_4_ hemisphere, possibly spanning a dynamic range of conformations which allows fluctuation between the canonical ^1^C_4_ chair and the B_03_ / ^1^S_3_ state.

## Conclusion

A new strategy is presented to prepare selective ^13^C‐isotope labeled C‐Man glycoproteins, which allows the use of advanced experiments for detailed NMR investigation of the glycans. The method is based on the development of a *Drosophila* S2 cell line which lacks the MPI enzyme such that mannose cannot be converted from glucose (and the other way around) and is therefore potentially also applicable to other kind of glycosylation modifications containing mannose (O‐ and N‐glycosylation) or fucose, which is derived from mannose. Notably, the *Drosophila* S2 cell line used in this work appeared to be especially suitable for the expression as yeast and mammalian cells show mannose toxicity after deletion of the MPI enzyme.[^40^, ^41^] We did not observe problems with the cell growth at least till 1 mm of mannose.

Preceding NMR[^33^, ^42^, ^43^] and X‐ray crystallography[^44^, ^45^, ^46^] studies suggested that C‐mannoses can occur both in ^4^C_1_ and ^1^C_4_ conformations. Therefore, we evaluated the conformation of C‐mannose by using the selective ^13^C‐ mannose labeled UNC‐5 TSR2. Thermal denaturation studies on the protein showed that C‐Man plays an important role in the protein stability (possibly by assisting the formation of the Trp‐Arg ladder) and simulations performed with C‐mannoses in either ^1^C_4_ and ^4^C_1_ chair arrangements indicated similar effects.^[24]^ Nevertheless, investigation of the mannose conformational space and dynamics may allow us to understand in more detail by what means the sugar geometry and orientations can allow (specific) interactions with surrounding residues and thereby affect the protein fold.

Using our novel labeling strategy, the UNC‐5 thrombospondin repeat was expressed at high yields with incorporation of uniform ^13^C‐isotope labeled mannose which allowed us to nicely resolve the signals of the mannose moieties (from the protein signals) and perform NMR experiments that require the presence of the ^13^C label. In particular, the selective ^13^C‐labeling enables the investigation of the C‐mannose dynamics by heteronuclear ^13^C‐relaxation experiments and more detailed structural conformational studies by using cross‐correlated relaxation and ^13^C‐resolved NOESY experiments. Investigation of the sugar dynamics indicated that the UNC‐5 C‐mannoses are mostly rigid and therefore likely integrated in the stable structure of the folded protein. This is well in agreement with the observed increased thermal stability of the C‐mannosylated protein in comparison with the unmodified protein. The analysis of our ^3^ *J*(H,H), CCR(CH,CH) and NOE data shows that the C‐mannose puckers span a range of conformational space in the ^1^C_4_ hemisphere allowing interconversion between the canonical ^1^C_4_ and the B_03_ / ^1^S_3_ state. This conformation differs from the free mannose, which is most stable in the ^4^C_1_ hemisphere, but is well in agreement with previous NMR studies and simulations and retains the tryptophan indole moiety in the preferential equatorial position.[^32^, ^33^] Moreover, theoretical studies indicated that the B_03_ boat is a stable conformation^[47]^ and has only a very low energy barrier to the ^4^C_1_ conformation^[7]^ thus allowing an escape towards the free form of the Mannose. In addition, the ^1^C_4_ chair conformation and the flexibility of the ring permits a rotation of 180° around the C_1_‐C_δ1_ axis^[18]^ which may either be required for the catalytic process of the C‐mannosyltransferase to attach the mannose and/or necessary to arrive at the correct orientation for stabilizing contacts and hydrogen bond formation during protein (re)folding.

The exact role and mechanism of the sugar structure will only be fully understood when incorporating these observations in the larger context of the biological functions of the protein and the relevant dynamical aspects of its structure and molecular interplay, which are supposed to be affected or regulated by glycosylation. Our study using the UNC‐5 thrombospondin repeat shows a striking difference of the pyranose ring conformation between the free form and the C‐linked mannose. Other targets and glycosylation modifications may share this feature but could also show a substantially different behavior.[^26^, ^27^] We foresee that the novel tool presented in this work allows for more detailed NMR studies on the glycan 3D structures and its interactions, thereby providing a valuable foundation to advance structural glycoprotein research.

## Conflict of interest

The authors declare no conflict of interest.

## Supporting information

As a service to our authors and readers, this journal provides supporting information supplied by the authors. Such materials are peer reviewed and may be re‐organized for online delivery, but are not copy‐edited or typeset. Technical support issues arising from supporting information (other than missing files) should be addressed to the authors.

Supplementary
