# Supplementary material for: NMR Spectroscopic Characterization of the C‐Mannose Conformation in a Thrombospondin Repeat Using a Selective Labeling Approach
Source: Angew Chem Int Ed Engl. 2020 Sep 3;59(46):20659–65. doi: 10.1002/anie.202009489 (PMC7692951; doi:10.1002/anie.202009489)
Supplement: Supplementary file 1 — Supplementary [file ANIE-59-20659-s001.pdf]

Angewandte Chemie
Eine Zeitschrift der Gesellschaft Deutscher Chemiker

Supporting Information

NMR Spectroscopic Characterization of the C-Mannose Conformation in a Thrombospondin Repeat Using a Selective Labeling Approach

*Hendrik R. A. Jonker, Krishna Saxena, Aleksandra Shcherbakova, Birgit Tiemann, Hans Bakker, and Harald Schwalbe**

anie_202009489_sm_miscellaneous_information.pdf

Supporting Information
©Wiley-VCH 2019
69451 Weinheim, Germany

Abstract: Despite the great interest in glycoproteins, structural information reporting on conformation and dynamics of the sugar moieties are limited. We present a new biochemical method to express proteins with glycans that are selectively labeled with NMR active nuclei. We report on the incorporation of ^{13}C-labeled mannose in the C-mannosylated UNC-5 thrombospondin repeat. The conformational landscape of the C-mannose sugar puckers attached to tryptophan residues of UNC-5 is characterized by interconversion between the canonical $^{1}C_{4}$ state and the B_{03} / $^{1}S_{3}$ state. This flexibility may be essential for protein folding and stabilization. We foresee that this versatile tool to produce proteins with selective labeled C-mannose can be applied and adjusted to other systems and modifications and potentially paves a way to advance glycoprotein research by unravelling the dynamical and conformational properties of glycan structures and their interactions.

DOI: 10.1002/anie.202009489

Table of Contents

Experimental Procedures	page 2	S2 cell culture and mutant generation
	page 3	Protein expression and purification
	page 3	NMR spectroscopy
	page 3	Structure calculations
Supplementary Table 1.	page 4	Dynamics of free mannose and C-mannose
Supplementary Figures 1A-D.	page 5	1A: Analysis of $^3J(H_1,H_2)$ for free α- and β-mannose
	page 5	1B: Analysis of $^3J(H_2,H_3)$ for free α- and β-mannose
	page 6	1C: Analysis of $^3J(H_3,H_4)$ for free α- and β-mannose
	page 6	1D: Analysis of $^3J(H_4,H_5)$ for free α- and β-mannose
Supplementary Figures 2A-D.	page 7	2A: Analysis of $^3J(H_1,H_2)$ and $CCR(C_1H_1,C_2H_2)$ for UNC-5 W8-c α-mannose
	page 7	2B: Analysis of $^3J(H_2,H_3)$ and $CCR(C_2H_2,C_3H_3)$ for UNC-5 W8-c α-mannose
	page 8	2C: Analysis of $^3J(H_3,H_4)$ and $CCR(C_3H_3,C_4H_4)$ for UNC-5 W8-c α-mannose
	page 8	2D: Analysis of $^3J(H_4,H_5)$ and $CCR(C_4H_4,C_5H_5)$ for UNC-5 W8-c α-mannose
Supplementary Table 2.	page 9	Experimental ^3J and CCR values
Supplementary Figure 3.	page 10	CCR from the quantitative Γ-HCCH experiment
Supplementary Figure 4.	page 11	Exploring the landscape of mannose sugar conformations
References	page 12	

Experimental Procedures

S2 cell culture and mutant generation

Drosophila S2 cells (ThermoFisher) were routinely grown in suspension culture flasks at 24 °C, shaking at 30 rpm. A stable clone expressing the single TSR2 of C. elegans UNC-5 (pMT-UNC-5-TSR2), having an N-terminal His6 tag, in presence of C. elegans DPY-19 has been described before [1]. The sequence of the obtained protein is LDGG**W**SS**W**SD WSACSSSCHR YRTRACTVPP PMNGGQPCFG DDLMTQECPA QLCTADSTGH HHHHH, in which the first two tryptophans (W5 and W8) are C-mannosylated. To establish an MPI knock out in this cell line, the CRISPR/Cas9 vector pAc-sgRNA-Cas9 (gift from Ji-Long Liu; Addgene plasmid # 49330) [2,3] with target oligo sequence GCTCCATGACTCGACCAACT(CGG) was co-transfected (1 to 1) with the vector pCoBlast (ThermoFisher) using Polyethylenimine (PEI, MW 40,000, Polysciences) transfection. Clones were selected with 10 µg/ml of blasticidin (Invivogen) in 96 well plates with untransfected S2 cells as feeder cells. After about 3 weeks, the feeder cells died and wells with single growing colonies were selected for sequencing of a 402 bp PCR fragment surrounding the mutation site. The used clone in this experiment has a single sequence with a 55 bp deletion resulting in a frameshift after Q151 with a stopcodon 11 amino acids after.

Protein expression and purification

The generated cell line was cultured in Xpress medium containing 100 µM of mannose at 24 °C. For labelling, cells were pelleted and taken up in 300 ml medium without mannose at a concentration of 5x106 cells/ml. Cells were then cultured for one hour, followed by replacement of the medium by medium containing 500 µM uniformly labelled ^{13}C mannose (Eurisotop). At day two, Expression of the protein was induced with 4 µM $CdCl_2$ and placed at 28 °C. The increased temperature favors production of the C-mannosylated form of the TSR [1]. At day three, the cells obtained a second dose of ^{13}C mannose (200 µM). The culture medium was then harvested at day five. Medium was further processed basically as described [1]. Dialyzed medium (cut of 3000 Da) was purified in two batches by 1 ml nickel affinity chromatography (His-Trap HP, Cytiva). The proteins were additionally purified by C18 chromatography (Xbridge TM Prep C18 5 µm, 10 × 50 mm column Waters), allowing to separate di-, mono- and, non-mannosylated TSR2 forms, in 3 batches. The combined dimannosylated fractions were again loaded on a 1 ml His-Trap column to reduce the volume and finally purified using a 25 ml Superdex-75 gel filtration column (Cytiva) run in 10 mM KPO_4 (pH 7.4) and 50 mM NaCl. The final production of the 7 Kd UNC-5 TSR2 was about 2 mg, based on A280 measurement.

NMR spectroscopy

NMR experiments were performed at 298 K on Bruker spectrometers (600, 800, 900 or 950 MHz) equipped with cryogenic probes. The free mannose (10 mM) and C-mannosylated UNC-5 protein (1.25 mM) samples were measured in NMR buffer (containing 10 mM potassium phosphate (pH 7.6), 50 mM NaCl in either 90% H2O / 10% D_2O or 100% D_2O) using 3 mm NMR tubes. The spectrometer was locked on D_2O. The chemical shifts were referenced to DSS (2,2-dimethyl-2-silapentane-5-sulfonic acid) as internal standard [4]. The NMR resonance assignment could be performed using a combination of NOESY, HSQC, COSY and/or TOCSY type spectra. 2D $^{1}H^{1}H$-NOESY (with 80 and 120 ms mixing time) and 2D $^{1}H^{1}H$-COSY experiments were measured on unlabeled samples in 100% D_2O. Furthermore, 2D $^{1}H^{1}H$-NOESY (with 50, 75, 100 and 125 ms mixing time) spectra were measured on the C-mannosylated UNC-5 protein sample in H_2O. In addition, several 3D $^{1}H^{1}H^{13}C$-NOESY-HSQC (in D_2O with 60, 80, 100 and 120 ms and in H_2O with 120 ms mixing time) spectra and a 4D $^{1}H^{13}C^{1}H^{13}C$-NOESY-HSQC (in D_2O, 100 ms mixing time) spectrum were measured on the selective ^{13}C-labeled C-mannosylated UNC-5 protein.
The heteronuclear ^{13}C-relaxation experiments (^{13}C-T_1, ^{13}C-$T_{1\rho}$ and $\{^{1}H\}$-^{13}C heteronuclear NOE) were performed at 600 MHz. The ^{13}C-T_1 and ^{13}C-$T_{1\rho}$ relaxation rates for free ^{13}C-labeled mannose were determined from a series of 12 spectra with delays of 100, 200, 400, 600, 800, 1000, 1200, 1400, 1600, 2000, 2800 and 3600 ms. For the selective ^{13}C-labeled C-mannosylated UNC-5 protein, the relaxation delays were 50, 100, 200, 300, 400, 600, 800, 1000, 1200, 1600, 2000 and 2400 ms for determining the ^{13}C-T_1 relaxation rates and 5, 10, 15, 20, 25, 30, 35, 40, 50, 60, 80 and 100 ms for determining the ^{13}C-$T_{1\rho}$ relaxation rates. The $\{^{1}H\}$-^{13}C heteronuclear NOEs were determined from the ratio of signal intensities measured with and without presaturation. The calculation of the dynamics parameters was performed as described by Ferner et al. [5] using the program Modelfree 4 [6,7] for which the models were selected as proposed by d'Auvergne and Gooley [8].
The quantitative 2D $^{1}H^{13}C$-Γ-HCCH experiments, for determination of the Cross-Correlated Relaxation rates (CCR) was essentially performed as described in Felli et al. [9]. The cross experiment was generally acquired with 3 or 4 times the number of scans of the reference experiment. To resolve overlap, a 3D $^{1}H^{13}C^{13}C$ version of the experiment was implemented by including evolution of the carbon chemical shift in the second INEPT step,
All NMR spectra were processed by using TopSpin version 3.2 (Bruker Biospin) and analyzed with SPARKY version 3.114 (T. D. Goddard and D. G. Kneller, University of California, San Francisco).

Structure calculations

Structures of the C-mannosylated tryptophan moieties were calculated with CNS 1.1 [10] using ARIA 1.2 [11,12] setup. The NOESY cross peaks were carefully inspected and manually picked and assigned. The obtained peak lists were used as input for the conventional iterative structure calculation approach with automated relaxation matrix calculation and NOE to distance calibration including correction for spin diffusion (to improve the accuracy of the distance restraints). Dihedral angle restraints (based on the ^{3}J(H,H) couplings and CCR(CH,CH) data) were validated and confirmed by calculations performed with only NOE data. The structure calculation (with adapted protocols and forcefield to include the C-mannose moiety) was performed using a four stage simulated annealing (SA) protocol with cartesian angle dynamics for 200 structures per iteration and 2000 structures in the last stage. The high temperature stage consisted of 10000 steps at 10000 K. This was followed by refinement and cooling down stages: 8000 steps at 2000 K, 20000 steps to 1000 K and 15000 steps to 50 K. During the SA protocol the force constant for the distance restraints was set to 0, 10, 10 and 50 kcalmol-1Å-2 for the successive stages. The top-ranked 1000 lowest energy structures were further refined [13] and analyzed.

Table S1. Dynamics of free mannose and C-mannose. Dynamics by heteronuclear ^{13}C-relaxation experiments (T_1, T_2 and HetNOE) and the determined order parameter (S^2) from model-free analysis. The errors are estimated from the signal/noise (and set to a minimum of 0.01 for the HEtNOE).

**c-Man W5**	T_1 (ms)	T_2 (ms)	HetNOE	S^2
C1-H1	703 ± 12	36.3 ± 1.1	1.27 ± 0.04	0.94 ± 0.04
C2-H2	784 ± 20	39.0 ± 1.3	1.31 ± 0.04	0.85 ± 0.04
C3-H3	789 ± 17	32.7 ± 1.1	1.26 ± 0.04	0.95 ± 0.04
C4-H4	811 ± 16	34.7 ± 1.0	1.24 ± 0.03	0.91 ± 0.04
C5-H5	714 ± 20	38.1 ± 2.2	1.32 ± 0.04	0.91 ± 0.06

**c-Man W8**	T_1 (ms)	T_2 (ms)	HetNOE	S^2
C1-H1	845 ± 16	29.5 ± 0.9	1.17 ± 0.04	0.92 ± 0.04
C2-H2	816 ± 12	33.1 ± 0.8	1.18 ± 0.03	0.95 ± 0.02
C3-H3	883 ± 11	30.7 ± 0.7	1.21 ± 0.03	0.95 ± 0.02
C4-H4	830 ± 12	33.6 ± 0.7	1.19 ± 0.02	0.94 ± 0.02
C5-H5	751 ± 10	34.5 ± 1.4	1.23 ± 0.03	0.96 ± 0.04

**α-Mannose**	T_1 (ms)	T_2 (ms)	HetNOE	S^2
C1-H1	1150 ± 13	669 ± 15	2.43 ± 0.01	0.29 ± 0.01
C2-H2	1122 ± 6	925 ± 28	2.65 ± 0.01	0.39 ± 0.01
C3-H3	1186 ± 14	1068 ± 39	2.61 ± 0.01	0.24 ± 0.02
C4-H4	1166 ± 5	825 ± 13	2.54 ± 0.01	0.32 ± 0.01
C5-H5	1173 ± 3	815 ± 18	2.54 ± 0.01	0.31 ± 0.01

**β-Mannose**	T_1 (ms)	T_2 (ms)	HetNOE	S^2
C1-H1	1282 ± 14	1014 ± 25	2.67 ± 0.01	0.28 ± 0.02
C2-H2	1177 ± 9	760 ± 27	2.63 ± 0.01	0.40 ± 0.01
C3-H3	1228 ± 4	1104 ± 32	2.64 ± 0.01	0.23 ± 0.02
C4-H4	1236 ± 5	845 ± 14	2.58 ± 0.01	0.31 ± 0.01
C5-H5	1247 ± 3	923 ± 25	2.54 ± 0.01	0.30 ± 0.01

Figure S1A. Analysis of $^3J(H_1,H_2)$ for free α- and β-mannose

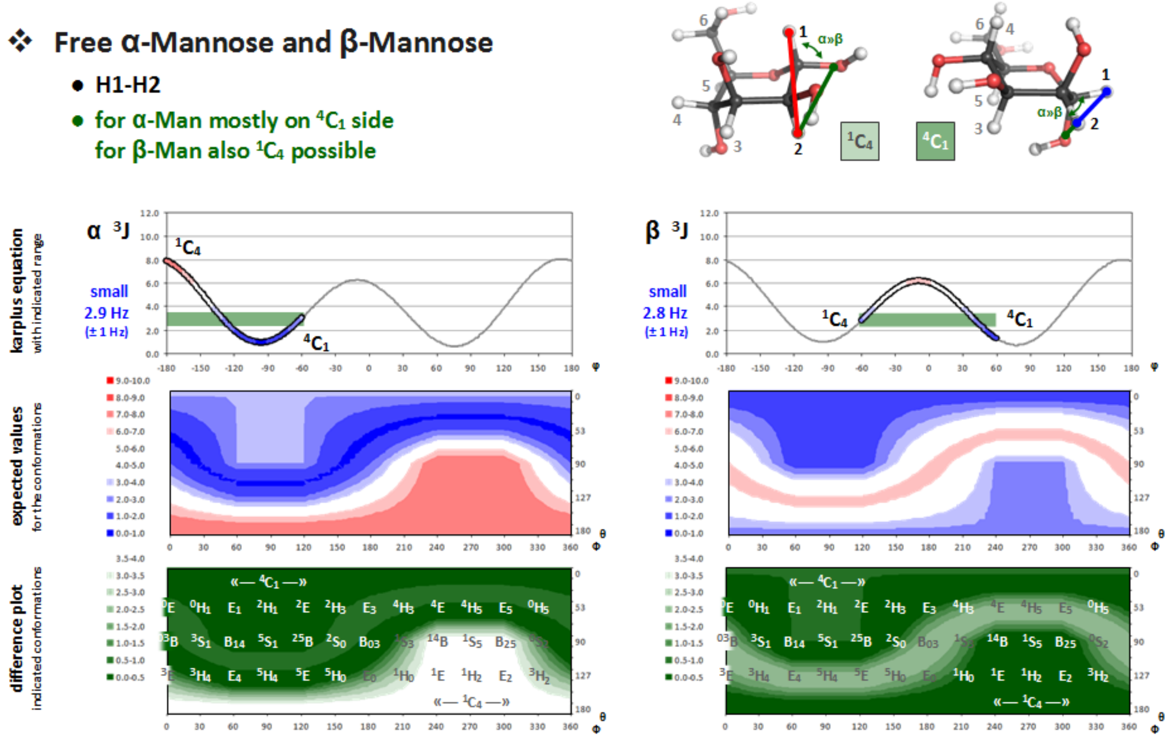

Figure S1B. Analysis of $^3J(H_2,H_3)$ for free α- and β-mannose

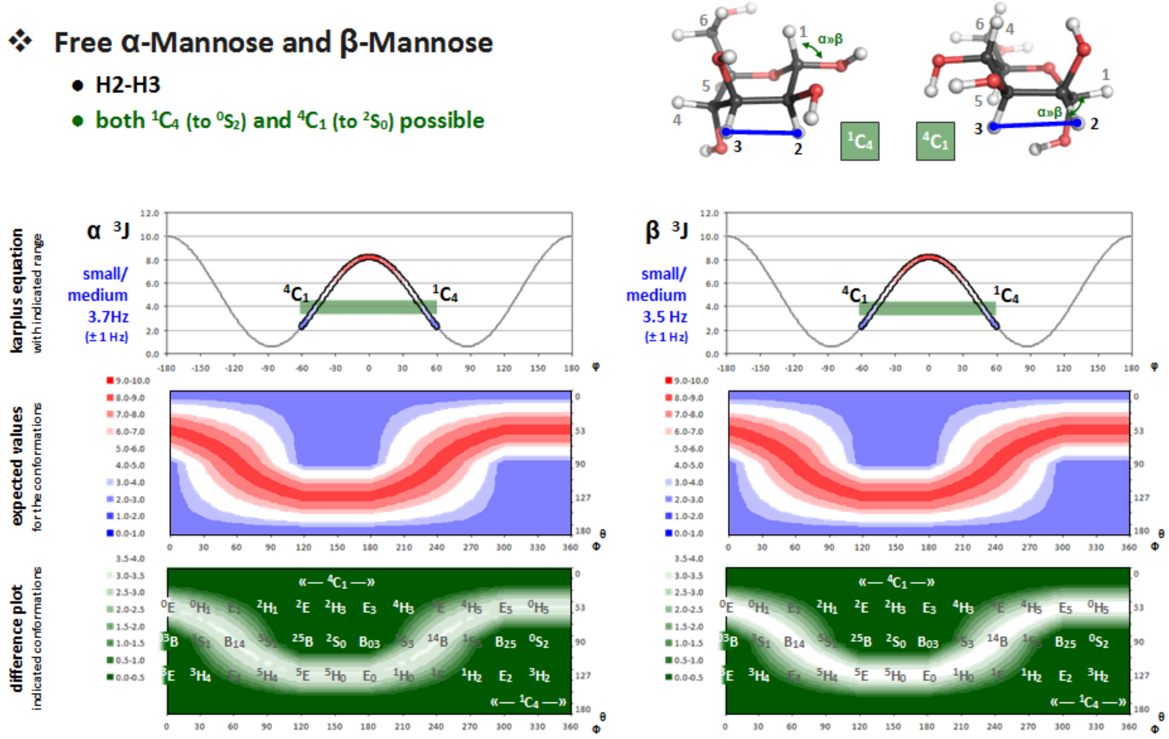

Figure S1C. Analysis of $^{3}J(H_3,H_4)$ for free α- and β-mannose

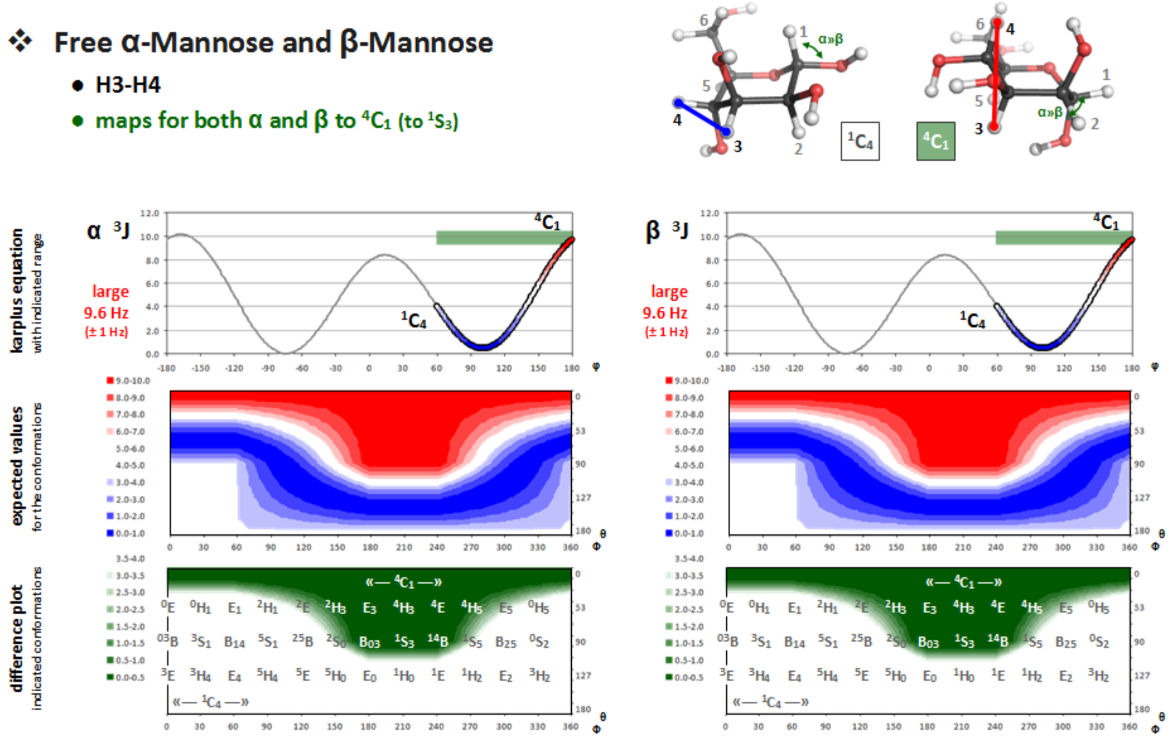

Figure S1D. Analysis of $^{3}J(H_4,H_5)$ for free α- and β-mannose

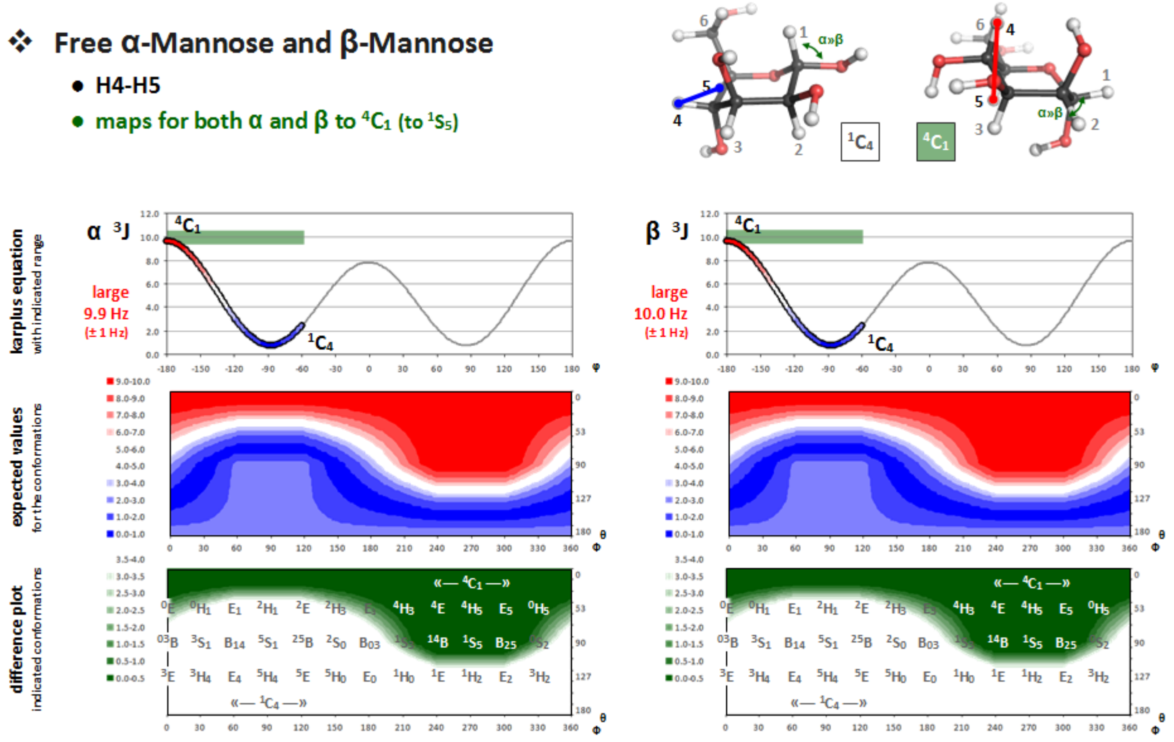

Figure S2A. Analysis of $^3J(H_1,H_2)$ and CCR(C_1H_1,C_2H_2) for UNC-5 W8-c α-mannose

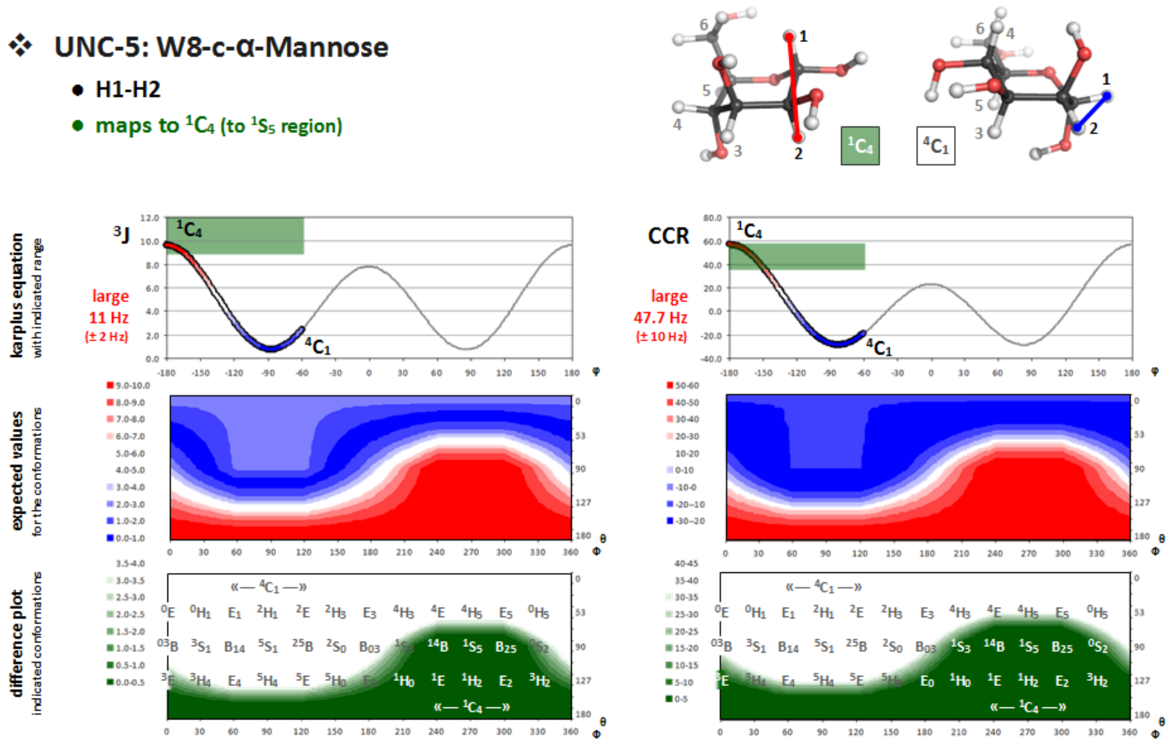

Figure S2B. Analysis of $^3J(H_2,H_3)$ and CCR(C_2H_2,C_3H_3) for UNC-5 W8-c α-mannose

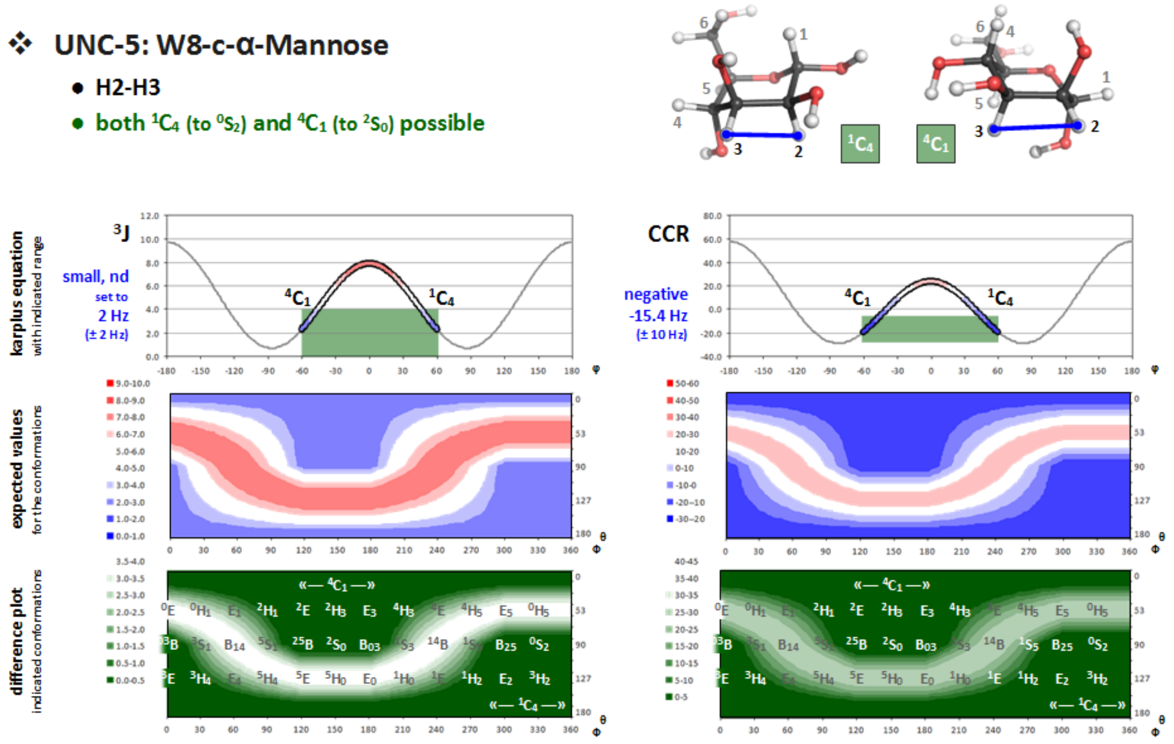

Figure S2C. Analysis of $^3J(H_3,H_4)$ and $CCR(C_3H_3,C_4H_4)$ for UNC-5 W8-c α-mannose

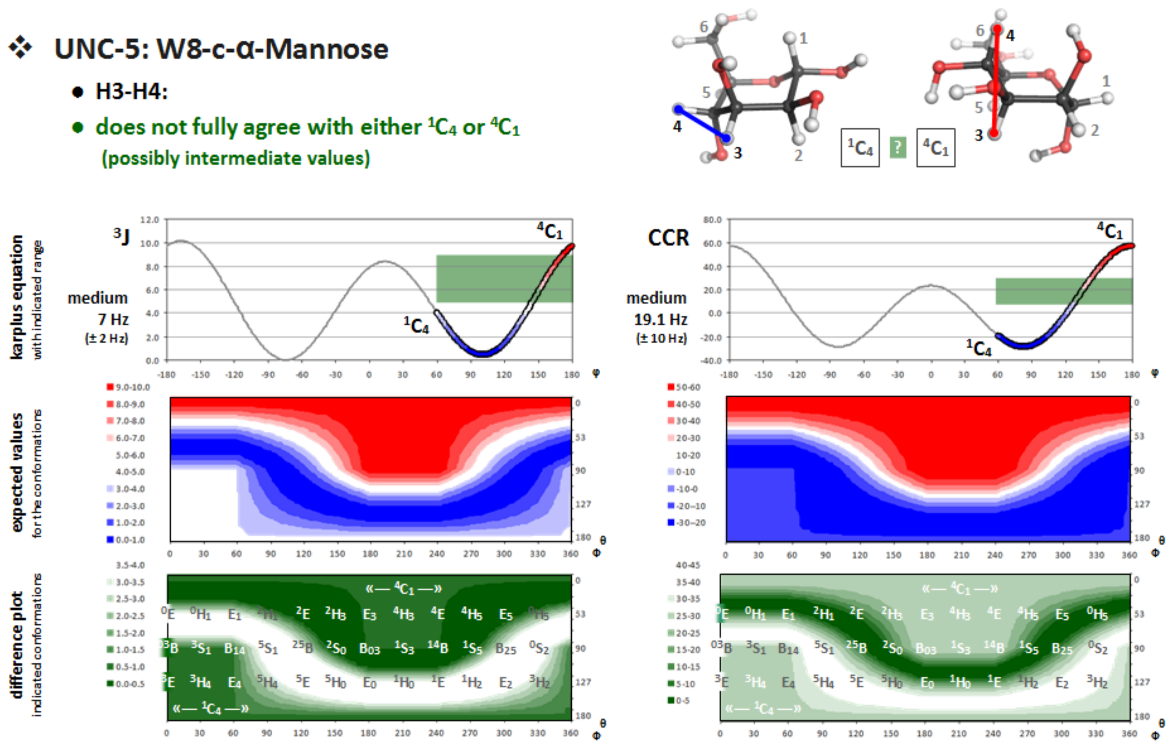

Figure S2D. Analysis of $^3J(H_4,H_5)$ and $CCR(C_4H_4,C_5H_5)$ for UNC-5 W8-c α-mannose

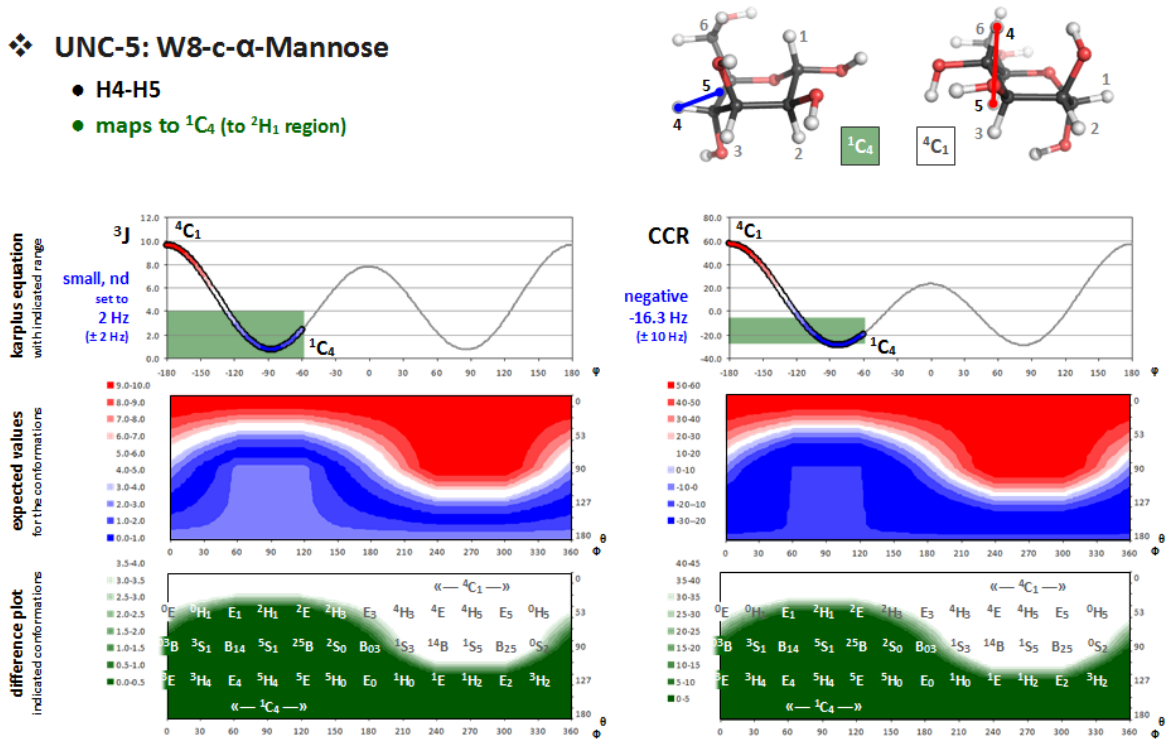

Table S2. Experimental ^{3}J and CCR values. ^{3}J(H,H) and CCR(CH,CH) values as extracted from 2D COSY and 2D and 3D Γ-HCCH experiments. The errors represent the deviation as used in the analysis (and are larger than the actual experimental errors as estimated from signal/noise). $^3J(H_2,H_3)$ and $^3J(H_4,H_5)$ values for c-Man were too small and could not be accurately determined (n.d.) and are therefore set to 2 ± 2 Hz.

**Free Mannose**	^{3}J (Hz) α	^{3}J (Hz) β
H1-H2	Small (2.9 ± 2)	Small (2.8 ± 2)
H2-H3	Small (3.7 ± 2)	Small (3.5 ± 2)
H3-H4	Large (9.6 ± 2)	Large (9.6 ± 2)
H4-H5	Large (9.9 ± 2)	Large (10.0 ± 2)

**UNC-5 c-Man W5**	^{3}J (Hz)	CCR (Hz)
H1-H2	Large (11.0 ± 2)	38.5 ± 10
H2-H3	Small (n.d., 2 ± 2)	-13.9 ± 10
H3-H4	Medium (6.5 ± 2)	27.5 ± 10
H4-H5	Small (n.d., 2 ± 2)	-18.0 ± 10

**UNC-5 c-Man W8**	^{3}J (Hz)	CCR (Hz)
H1-H2	Large (11.0 ± 2)	47.7 ± 10
H2-H3	Small (n.d., 2 ± 2)	-15.4 ± 10
H3-H4	Medium (7.0 ± 2)	19.1 ± 10
H4-H5	Small (n.d., 2 ± 2)	-16.3 ± 10

Figure S3. CCR from the quantitative Γ-HCCH experiment. The 2D $^{1}H^{13}C$ quantitative Γ-HCCH reference (left) and cross (right) experiment showing the Mannose C_1H_1 to C_5H_5 sugar region (and indicated walk) of the selective ^{13}C-labeled C-mannoses which are covalently attached to the tryptophans 5 (black) and 8 (grey) in the UNC-5 protein. For clarity, the spectra are shown with a different threshold (1:2). The carbon-proton dipole-dipole cross-correlation rates can be determined from the signal intensity ratio of the cross peaks [9].

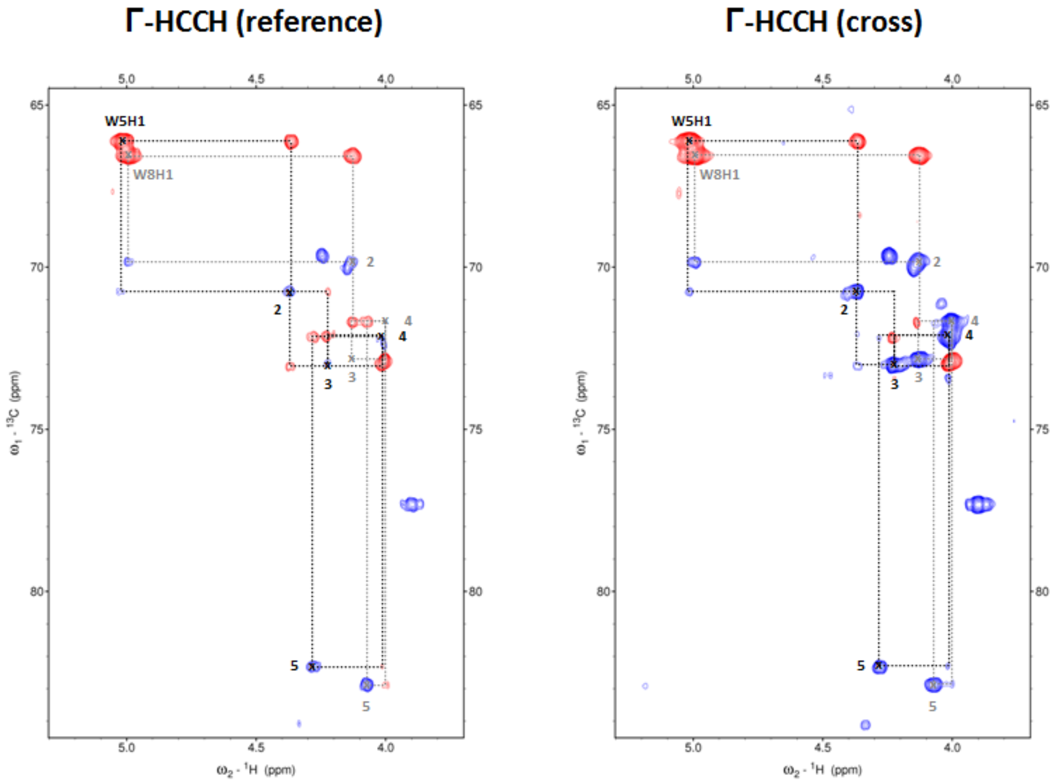

Figure S4. Exploring the landscape of mannose sugar conformations. The conformational landscape of the UNC-5 W5 and W8 C-α-mannose, as indicated by 3J(H,H), CCR(CH,CH) and NOE restrained structure calculations, indicated on the 2D Mercator projection map of the 'Cremer Pople' sphere, illustrating the regions of favorable sugar geometries. The combination of differences for the $^3J(H,H)$ and CCR(CH,CH) values is here examined without the intermediate $^3J(H_3,H_4)$ and $CCR(C_3H_3,C_4H_4)$ values. For each conformation the difference of the experimental values from the theoretical expected values (Karplus equation) was calculated and combined according to: $\sum\Delta=0.5\sqrt{(\Delta^2_{H1H2}+\Delta^2_{H2H3}+\Delta^2_{H4H5})}$. The conformational landscape resembles the one including all values (Main figure 3), spanning a likely range in the $^1C_4$ hemisphere. The CP puckering angles from the calculated ensemble of 1000 top-ranked lowest energy structures was extracted using scripts from Hill and Reilly [14] and demonstrate the favorable $^1C_4$ and B_{03} / $^1S_3$ conformational states.

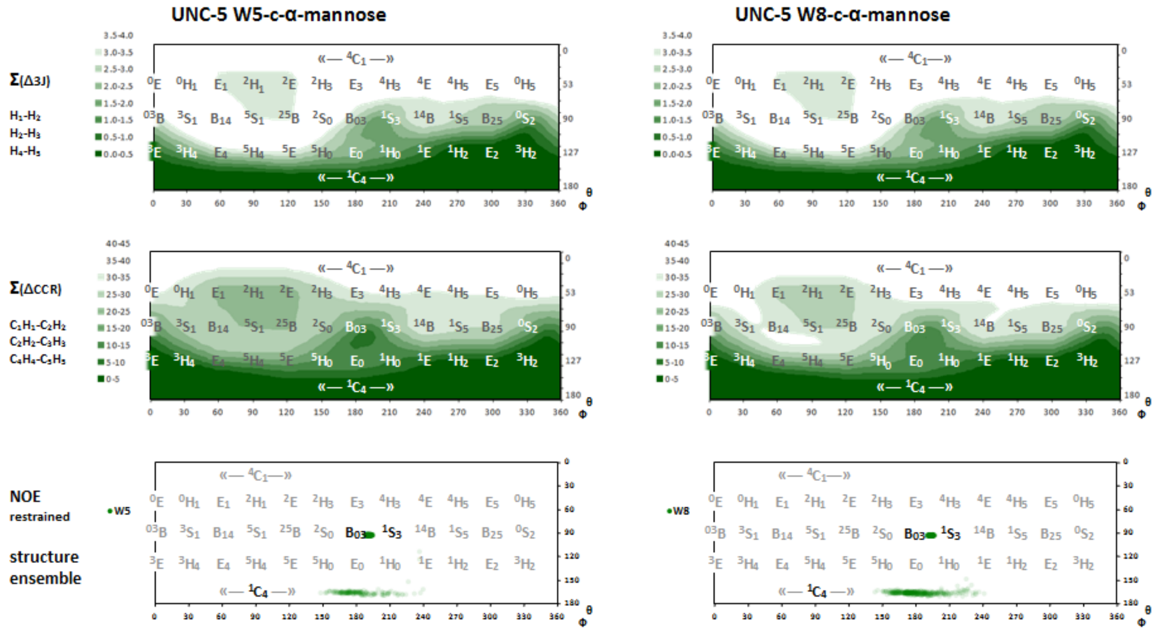

References

[1] A. Shcherbakova, M. Preller, M. H. Taft, J. Pujols, S. Ventura, B. Tiemann, F. F. R. Buettner, H. Bakker, *Elife* **2019**, *8*, DOI 10.7554/eLife.52978.

[2] A. R. Bassett, J. L. Liu, *J. Genet. Genomics* **2014**, *41*, 7–19.

[3] A. Bassett, J. L. Liu, *Methods* **2014**, *69*, 128–136.

[4] D. S. Wishart, *Prog. Nucl. Magn. Reson. Spectrosc.* **2011**, *58*, 62–87.

[5] J. Ferner, A. Villa, E. Duchardt, E. Widjajakusuma, J. Wöhnert, G. Stock, H. Schwalbe, *Nucleic Acids Res.* **2008**, *36*, 1928–1940.

[6] A. M. Mandel, M. Akke, A. G. Palmer, *J. Mol. Biol.* **1995**, *246*, 144–163.

[7] A. G. Palmer, M. Rance, P. E. Wright, *J. Am. Chem. Soc.* **1991**, *113*, 4371–4380.

[8] E. J. d'Auvergne, P. R. Gooley, *J. Biomol. NMR* **2003**, DOI 10.1023/A:1021902006114.

[9] I. C. Felli, C. Richter, C. Griesinger, H. Schwalbe, *J. Am. Chem. Soc.* **1999**, *121*, 1956–1957.

[10] A. T. Brünger, P. D. Adams, G. M. Clore, W. L. Delano, P. Gros, R. W. Grossekunstleve, J. S. Jiang, J. Kuszewski, M. Nilges, N. S. Pannu, R. J. Read, L. M. Rice, T. Simonson, G. L. Warren, *Acta Crystallogr. Sect. D Biol. Crystallogr.* **1998**, *54*, 905–921.

[11] J. P. Linge, M. Habeck, W. Rieping, M. Nilges, *Bioinformatics* **2003**, *19*, 315–316.

[12] J. P. Linge, S. I. O'Donoghue, M. Nilges, *Methods Enzymol.* **2001**, *339*, 71–90.

[13] J. P. Linge, M. A. Williams, C. A. E. M. Spronk, A. M. J. J. Bonvin, M. Nilges, *Proteins Struct. Funct. Genet.* **2003**, *50*, 496–506.

[14] A. D. Hill, P. J. Reilly, *J. Chem. Inf. Model.* **2007**, *47*, 1031–1035.
